# Prognostic value of procalcitonin in respiratory tract infections across clinical settings

**DOI:** 10.1186/cc14145

**Published:** 2015-03-16

**Authors:** A Kutz, B Mueller, P Schuetz, for the IPD Study Group

**Affiliations:** 1 Kantonsspital Aarau, Switzerland

## Introduction

Whether the inflammatory biomarker procalcitonin (PCT) provides prognostic information across clinical settings and different acute respiratory tract infections (ARI) is poorly understood. Herein, we investigated the prognostic value of admission PCT levels to predict adverse clinical outcome in a large ARI population.

## Methods

We analyzed data from 14 trials and 4,211 ARI patients to study associations of admission PCT levels and setting specific treatment failure and mortality alone at 30 days. We used multivariable hierarchical logistic regression and conducted sensitivity analyses stratified by clinical settings and ARI diagnoses to assess the results' consistency.

## Results

**Figure 1:**
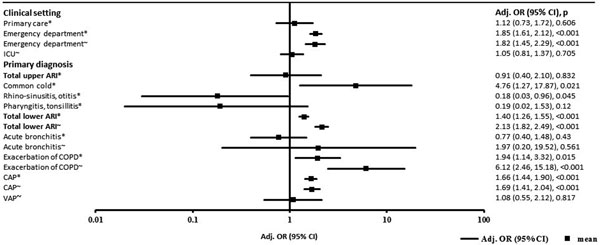
**Multivariate regression analysis for estimation of predictive value of PCT levels on admission stratified by adverse events and mortality in different settings and diagnoses**. *Treatment failure, ~mortality. ARI, acute respiratory tract infection; ECOPD, exacerbated chronic obstructive pulmonary disease; CAP, community-acquired pneumonia; VAP, ventilator-associated pneumonia; Adj., adjusted; OR, odds ratio; CI, confidence interval.

Overall, 864 patients (20.5%) experienced treatment failure and 252 (6.0%) died. The ability of PCT to differentiate patients with and without treatment failure was highest in the emergency department setting (treatment failure; area under the curve (AUC): 0.64 (95% confidence interval (CI): 0.61, 0.67), adjusted odds ratio (OR): 1.85 (95% CI: 1.61, 2.12), *P *< 0.001 - mortality; AUC: 0.67 (95% CI: 0.63, 0.71), adjusted OR: 1.82 (95% CI: 1.45, 2.29), *P *< 0.001). In lower respiratory tract infections, PCT was a good predictor of identifying patients at risk for mortality (AUC: 0.71 (95% CI: 0.68, 0.74), adjusted OR: 2.13 (95% CI: 1.82, 2.49), *P *< 0.001). In primary care and ICU patients no significant associations of initial PCT levels and outcome were found. See Figure 1.

## Conclusion

Admission PCT levels are associated with setting specific treatment failure and provide most prognostic information in ARI in the emergency department setting.
